# ^1^H, ^13^C, and ^15^N resonance assignment of photoactive yellow protein

**DOI:** 10.1007/s12104-012-9387-9

**Published:** 2012-04-19

**Authors:** Trijntje J. Pool, Nur Alia Oktaviani, Hironari Kamikubo, Mikio Kataoka, Frans A. A. Mulder

**Affiliations:** 1 Groningen Biomolecular Sciences and Biotechnology Institute, University of Groningen, Nijenborgh 4, 9747 AG Groningen, The Netherlands; 2 Graduate School of Materials Science, Nara Institute of Science and Technology, 8916-5 Takayama, Ikoma, Nara, 630-0192 Japan; 3 Present Address: Department of Chemistry, Interdisciplinary Nanoscience Center (iNANO), University of Aarhus, Langelandsgade 140, 8000 Aarhus C, Denmark

**Keywords:** PYP, *Halorhodospira halophila*, *para*-coumaric acid, NMR spectroscopy, Photoactivation

## Abstract

Photoactive yellow protein (PYP) is involved in the negative phototactic response towards blue light of the bacterium *Halorhodospira halophila*. Here, we report nearly complete backbone and side chain ^1^H, ^13^C and ^15^N resonance assignments at pH 5.8 and 20 °C of PYP in its electronic ground state.

## Biological context

Photoactive yellow protein (PYP) is a 125 amino acid (14 kDa) water-soluble, blue-light sensor protein, first found in the halophilic bacterium *Halorhodospira halophila* (Meyer 1985). PYP is a photoreceptor, believed to be responsible for the negative phototactic response of its host organism (Sprenger et al. 1993). This kind of response is required for organisms to evade potentially harmful short-wavelength light. Based on this observation, PYP has become a suitable model to understand the signal-transduction mechanism in Per-Arnt-Sim (PAS) domain signaling (Crosthwaite et al. 1997; Nambu et al. 1991). Several PYP-like proteins have meanwhile been found in other organisms, where they are also thought to act as light sensors. In addition, PYP-like proteins found in purple bacteria are involved in cell buoyancy or sensing bacteriophytochromes (Jiang et al. 1999; Kyndt et al. 2004).

Understanding of light transduction in PYP requires structural information in atomic detail. A 1.4 Å crystallographic structure was determined in 1995 by Borgstahl et al. and in 1998 Düx and coworkers revealed the solution structure and backbone dynamics of PYP by NMR spectroscopy. The reaction center of PYP is protected from solvent by R52, which is believed to function as a gateway in the photocycle (Borgstahl et al. 1995; Genick et al. 1997). The chromophore, *para*-coumaric acid (*p*CA), is covalenty bound to C69 with a thioester bond and *p*CA participates in two short hydrogen bonds with E46 and Y42 to stabilize the negative charge of *p*CA in the electronic ground state, pG. Upon blue-light capture, the chromophore undergoes *trans*–*cis* isomerisation and the intermediate pR is formed, which subsequently relaxes to the proposed signaling state, pB. In the latter state, the reaction center is exposed and the two short hydrogen bonds are broken (Borgstahl et al. 1995; Sigala et al. 2009; Yamaguchi et al. 2009).

In this paper, we present the nearly complete assignment of the backbone and side chain resonances of the pG state of PYP.

## Methods and experiments

Uniformly ^13^C,^15^N-labeled wild type PYP was overexpressed and purified as described previously (Düx et al. 1998). The NMR sample contained 1.2 mM doubly labeled [^13^C, ^15^N] PYP, 5 mM potassium phosphate buffer pH 5.8, 10 % D_2_O and 0.15 mM 2,2-dimethyl-2-silapentane-5-sulfonic acid (DSS). All 1D ^1^H, 2D and 3D NMR experiments were carried out at 20 °C using a Varian Unity INOVA 600 MHz spectrometer equipped with a field-gradient probe. Referencing was performed according to the method described by Wishart et al. (1995) using DSS. To obtain the sequential backbone resonance assignments, 2D ^15^N-HSQC equipped with sensitivity enhancement and water flip back pulses (Kay et al. 1992; Zhang et al. 1994), 3D CBCA(CO)NH/HNCACB (Wittekind and Mueller 1993) and 3D HNCO/HN(CA)CO (Ikura et al. 1990; Yamazaki et al. 1994) experiments were used.

To accomplish most of the side chain assignments, the strategy outlined by Oktaviani, et al. (2011) was performed. 3D (H)C(CO)NH-TOCSY and H(CCO)NH-TOCSY were used to assign carbon and proton aliphatic side chain resonances, respectively (Grzesiek et al. 1993; Logan et al. 1993; Montelione et al. 1992). For aromatic side chains, CB(CGCD)HD, CB(CGCDCE)HE (Yamazaki et al. 1993a), ^1^H–^13^C HSQC CParo (Zuiderweg et al. 1996), 2D ^13^C-^1^H CT HSQC, and 2D ^13^C–^1^H CT HSQCaro were recorded. Aromatic C^γ^–H^β^ correlations were detected using CG(CB)HB experiments (Prompers et al. 1998). Carbonyl ^13^C side chain resonances of Asx and Glx residues were assigned using H2(C)CO (Kay et al. 1990; Powers et al. 1991; Yamazaki et al. 1993b) and (HBGCBG)CO(CBGCABCON)H (Tollinger et al. 2002) experiments. N^ζ^–H^ε^ (Lys) and N^ε^–H^δ^ (Arg) correlations were detected using H2(C)N (André et al. 2007).

All spectra were processed using NMRPipe (Delaglio et al. 1995) and they were analyzed with Sparky (Goddard and Kneller 2008).

## Completeness of assignments and data deposition

**Fig. 1:**
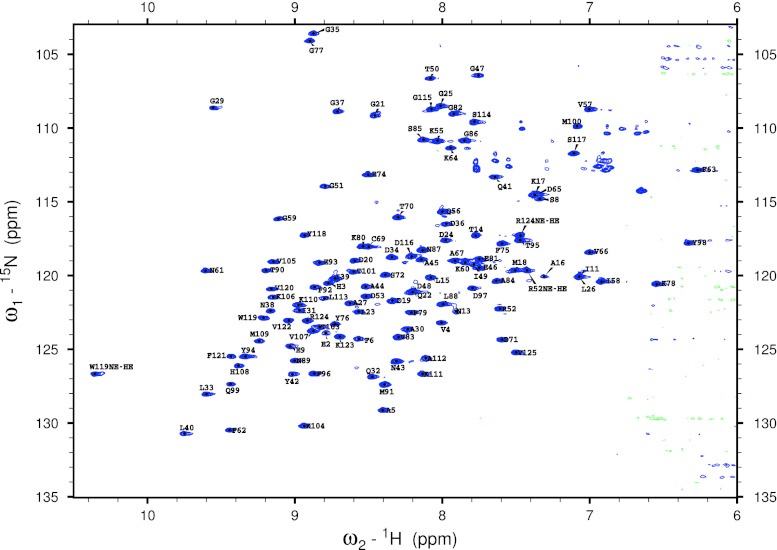
2D ^15^N-HSQC spectrum of PYP at pH 5.8 and 20 °C

The obtained backbone assignment is over 96 % complete (see Fig. 1). Missing assignments are the amide backbone ^15^N frequencies of the four Pro residues, and the amide nitrogen and proton frequencies of M1 and G7. At this pH no backbone assignment was possible for E12 and the ^13^C’ frequencies of F6, D10 and I11 were not found.

The entire ^1^H, ^13^C and ^15^N side chain resonance assignment is over 85 % complete. Unassigned side chain resonances mainly pertain to labile protons and their attached heteroatoms, such as N^η^/H^η^ of the two Arg residues, protons connected to oxygen (i.e. Tyr, Ser and Thr OH), protons bound to nitrogen and their corresponding nitrogens in the imidazole moiety of His residues, H^ζ^ of Lys, and also resonances due to several non-labile groups, such as methyl C^ε^/H^ε^ of Met residues, and a number of signals due to Phe and Trp ring systems.

**Fig. 2:**
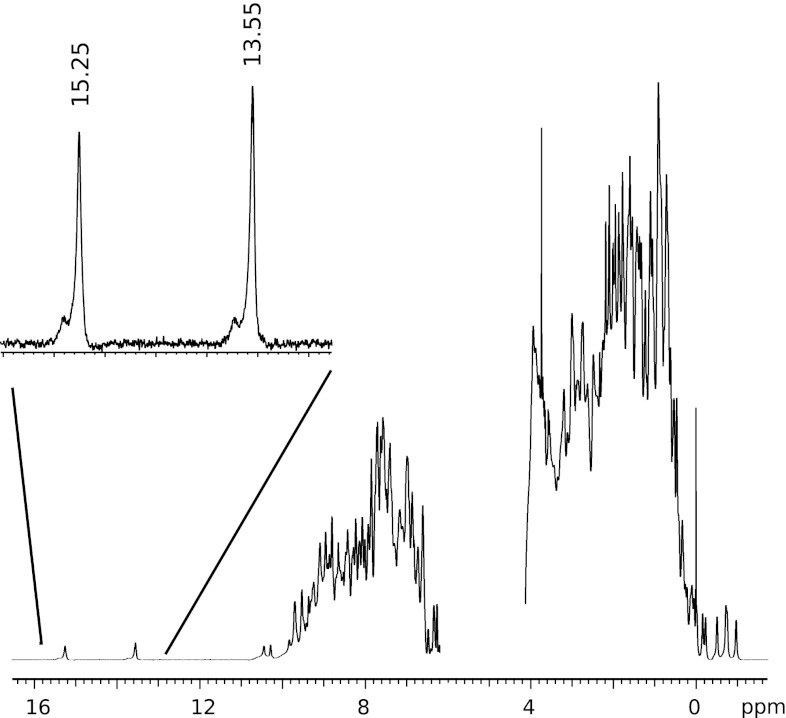
1D ^1^H NMR spectrum of PYP at pH 5.8, obtained with a selective water flip-back pulse, to detect the hydrogen bonds donated by Y42 and E46 to the chromophore oxygen. The downfield region is enlarged and the hydroxyl resonances of E46 and Y42 are observed at 15.25 and 13.55 ppm, respectively

The C^ζ^ frequency of the tyrosine residues were found in the ^1^H–^13^C HSQC CParo experiment (run overnight) and could be assigned by using a combination of CB(CGCD)HD and CB(CGCDCE)HE spectra. However, Y76 and Y118 have too similar H^ε^ frequencies to be able to determine which H^ε^-C^ζ^ peak corresponds to which residue. Therefore this information was obtained from pH-titration experiments, using the fact that tyrosine C^γ^ and C^ζ^ chemical shifts change simultaneously by large amounts during this titration. As the Y76 signals start moving at a lower pH than those of Y118, it was possible to assign the C^ζ^ nuclei in this fashion. We also can detect signals due to two short hydrogen bonds, which belong to Y42 and E46, using a 1D proton water flip-back sequence that suppresses the water signal without saturating it (see Fig. 2). These NMR signals have been assigned by Sigala et al*.* (2009).

Previously, ^1^H and ^15^N chemical shift assignments have been made at 37 °C by Düx et al*.* (1998), but these have not been submitted to the BMRB. Our results agree well with these, with a few exceptions. ^1^H, ^13^C and ^15^N assignments for the N-terminal deletion variant Δ25-PYP at 20 °C are available under BMRB accession number 6321 (Bernard et al. 2005), and these show significant differences as a result of the removal of part of the native protein structure.

The ^1^H, ^13^C and ^15^N assignment can be found in the BioMagResBank under accession number 18122.
